# 

**Published:** 1911-01-21

**Authors:** 


### THE HOSPITAL

Telegraphic Address: Ospedale, London.

THE MODERN MEDICAL AND INSTITUTIONAL JOURNAL.

Telephone Number: 2734 Gerrard.

NEW SERIES. No. 207, Vol. VIII.
No. 1279, Vol. XLIX.

SATURDAY, JAN. 21ST, 1911.

PRICE THREEPENCE

